# An international multicentre evaluation of treatment strategies for combined hepatocellular-cholangiocarcinoma^✰^^✰^

**DOI:** 10.1016/j.jhepr.2023.100745

**Published:** 2023-03-28

**Authors:** Marco P.A.W. Claasen, Tommy Ivanics, Berend R. Beumer, Roeland F. de Wilde, Wojciech G. Polak, Gonzalo Sapisochin, Jan N.M. IJzermans

**Affiliations:** 1 Multi-Organ Transplant Program, University Health Network, Toronto, Ontario, Canada; 2 Department of Surgery, Division of HPB & Transplant Surgery, Erasmus MC Transplant Institute, University Medical Centre Rotterdam, Rotterdam, The Netherlands; 3 Department of Surgery, Henry Ford Hospital, Detroit, MI, USA; 4 Department of Surgical Sciences, Akademiska Sjukhuset, Uppsala University, Uppsala, Sweden; 5 Division of General Surgery, University Health Network, Toronto, Ontario, Canada

**Keywords:** Mixed hepatocellular cholangiocarcinoma, Hepatocholangiocarcinoma, Cholangiolocarcinoma, Biphenotypic, Intermediate cell, Biliary tract cancer, Liver cancer, Hepatocellular carcinoma, HCC, CCA, Therapeutic management, Treatment policies

## Abstract

**Background & Aims:**

Management of combined hepatocellular-cholangiocarcinoma (cHCC-CCA) is not well-defined. Therefore, we evaluated the management of cHCC-CCA using an online hospital-wide multicentre survey sent to expert centres.

**Methods:**

A survey was sent to members of the European Network for the Study of Cholangiocarcinoma (ENS-CCA) and the International Cholangiocarcinoma Research Network (ICRN), in July 2021. To capture the respondents’ contemporary decision-making process, a hypothetical case study with different tumour size and number combinations was embedded.

**Results:**

Of 155 surveys obtained, 87 (56%) were completed in full and included for analysis. Respondents represented Europe (68%), North America (20%), Asia (11%), and South America (1%) and included surgeons (46%), oncologists (29%), and hepatologists/gastroenterologists (25%). Two-thirds of the respondents included at least one new patient with cHCC-CCA per year. Liver resection was reported as the most likely treatment for a single cHCC-CCA lesion of 2.0–6.0 cm (range: 73–93%) and for two lesions, one up to 6 cm and a second well-defined lesion of 2.0 cm (range: 60–66%). Nonetheless, marked interdisciplinary differences were noted. Surgeons mainly adhered to resection if technically feasible, whereas up to half of the hepatologists/gastroenterologists and oncologists switched to alternative treatment options with increasing tumour burden. Fifty-one (59%) clinicians considered liver transplantation as an option for patients with cHCC-CCA, with the Milan criteria defining the upper limit of inclusion. Overall, well-defined cHCC-CCA treatment policies were lacking and management was most often dependent on local expertise.

**Conclusions:**

Liver resection is considered the first-line treatment of cHCC-CCA, with many clinicians supporting liver transplantation within limits. Marked interdisciplinary differences were reported, depending on local expertise. These findings stress the need for a well-defined multicentre prospective trial comparing treatments, including liver transplantation, to optimise the therapeutic management of cHCC-CCA.

**Impact and implications:**

Because the treatment of combined hepatocellular-cholangiocarcinoma (cHCC-CCA), a rare form of liver cancer, is currently not well-defined, we evaluated the contemporary treatment of this rare tumour type through an online survey sent to expert centres around the world. Based on the responses from 87 clinicians (46% surgeons, 29% oncologists, 25% hepatologists/gastroenterologists), representing four continents and 25 different countries, we found that liver resection is considered the first-line treatment of cHCC-CCA, with many clinicians supporting liver transplantation within limits. Nonetheless, marked differences in treatment decisions were reported among the different specialties (surgeon *vs.* oncologist *vs*. hepatologist/gastroenterologist), highlighting the urgent need for a standardisation of therapeutic strategies for patients with cHCC-CCA.

## Highlights

• Liver resection is considered the first-line treatment of cHCC-CCA.
• Many clinicians support liver transplantation as a treatment option for cHCC-CCA.
• Marked interdisciplinary differences in treatment strategies exist globally.
• Well-defined cHCC-CCA treatment policies are lacking at most centers.
• Standardization of therapeutic strategies for cHCC-CCA is needed.

## Graphical abstract

**Figure:**
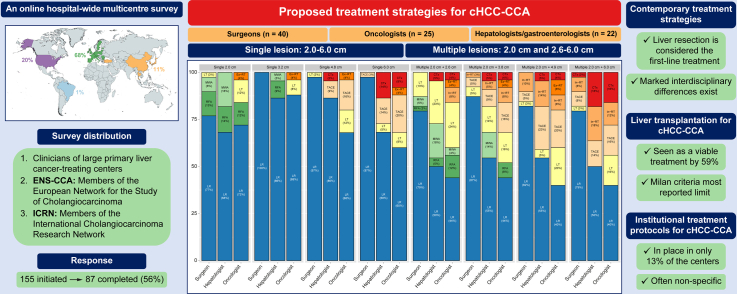

## Introduction

Combined hepatocellular-cholangiocarcinoma (cHCC-CCA) is a rare tumour with an incidence of 0.59 per 1,000,000 individuals.^1^ It shares histopathological features of both hepatocellular carcinoma (HCC) and cholangiocarcinoma (CCA) and represents roughly 1% of all primary liver cancers.^2^^,^^3^ After Lisa and Allen introduced the first histopathologic classification for this tumour type in 1949,^4^ the number of studies reporting on cHCC-CCA treatment outcome has been limited. The studies that published outcomes after different treatments for cHCC-CCA were characterised by small numbers, differently defined types of cHCC-CCA over time, and conflicting results.[5], [6], [7], [8] Moreover, because of an absence of effective tools for diagnosing this rare tumour type, many of these patients were initially misdiagnosed as having HCC, only to find out post-treatment that it turned out to be cHCC-CCA.^9^ Altogether, this has resulted in different treatment strategies for cHCC-CCA being applied across primary liver cancer-treating centres worldwide.^10^ We aimed to map these differences and use the insight to accelerate the development of a universal approach to improve the outcome of patients with cHCC-CCA.

## Patients and methods

This study complied with the guidelines for human studies and was conducted in accordance with the World Medical Association Declaration of Helsinki. The study was approved by the Medical Ethics Committee of Erasmus MC, University Medical Centre Rotterdam (MEC-2021-0204).

### Study objectives

This study aimed to identify the contemporary hospital-wide approach for patients with cHCC-CCA worldwide by capturing the experiences of clinicians working in primary liver cancer-treating centres. To successfully map this, the following areas were evaluated:

1. Respondent demographics;
2. Centre specifics related to primary liver cancer treatment;
3. cHCC-CCA occurrence and pre-treatment diagnosis rate;
4. Perceptions of and experiences with cHCC-CCA outcomes;
5. Patient-specific treatment recommendations for cHCC-CCA;
6. Centre-specific treatment policies related to cHCC-CCA treatment.

In addition, the study sought to contextualise the identified hospital-wide treatment strategies with what is known in the current literature about their efficacy and post-treatment outcomes.

### Study population

By design, all treating clinicians working at international primary liver cancer-treating centres were considered eligible for study participation.

### Survey development and design

A dedicated web-based survey environment known as LimeSurvey® was used to develop and process the survey. The survey was designed and structured based on the previously highlighted areas of interest. To ensure that the purpose of the study would be met, all essential questions required completion before moving on to the next section of the survey. Non-essential questions were subject to the same requirement but had additional response options to indicate that the question could not be answered or that the answer was unknown to the respondent. As a result, after survey completion, no question would remain unanswered, thus avoiding missing data. If a respondent’s department did not treat patients with cHCC-CCA and had not encountered any such patients in the past 5 years, the survey was terminated after answering the questions related to the respondent’s demographics and centre specifics. This, too, was considered survey completion. The full survey can be found in the Supplementary material.

### Patient-specific treatment recommendations for cHCC-CCA

To gain insight into contemporary treatment strategies, overall and per discipline, a hypothetical case study was embedded in the survey to ask respondents which treatment they would propose for varying combinations of tumour size and number. These varying combinations were grouped into two main scenarios. One in which the patient had a single lesion ranging between 2.0 and 6.0 cm (segment 3), and another in which the patient had two lesions, one with a static size of 2.0 cm (segment 2), and one with a size varying between 2.6 and 6.0 cm (segment 3). The hypothetical case to which all these scenarios related consisted of a 53-year-old female patient with an unremarkable medical history, a biopsy-proven, well-differentiated cHCC-CCA (based on biopsy of the largest lesion), with no signs of extrahepatic disease or vascular invasion, a Child–Pugh score of A6, and with all detected lesions being deemed resectable. All tumour size and number combinations asked for can be found in the full survey (Supplementary material) and are reported in the results section.

### Survey distribution

To obtain an international, multidisciplinary response from predominantly medium- to large-sized hospitals treating primary liver cancers, and to ensure a globally diverse representation, the survey was distributed through three different channels:

1. The European Network for the Study of Cholangiocarcinoma (ENS-CCA);
2. The International Cholangiocarcinoma Research Network (ICRN);
3. A region-based selected group of clinicians working at large primary liver cancer-treating centres worldwide.

To maximise response rates, distribution through each channel consisted of (1) an initial email invitation to participate in the survey; and (2) a follow-up email after 2 months reminding participants to complete the survey if they had not already done so. The survey was first distributed in June 2021 and last shared by November 2021. The study closed on December 1, 2021.

### Consent

To ensure that participants would consent to provide information for this study, the survey’s first question was dedicated to this cause. If no consent was given, the survey was terminated, and no data related to these participants were recorded or used for this study. Only data of participants who provided consent were analysed.

### Data handling

Given that completion of each section of the survey was imperative to provide a clear view of the hospital-wide approach to cHCC-CCA, and the inclusion of responses from incompletely answered surveys may result in distorted findings, it was decided not to include incomplete surveys in the analyses. In the case of duplicate entries, only the first complete survey response was included. Surveys from non-patient-treating clinicians and full-time researchers were excluded from the analysis. There was no missing data as the study design made it impossible to leave questions unanswered.

### Contextualisation identified hospital-wide treatment strategies

To evaluate how the identified hospital-wide treatment strategies compared to the evidence in the current literature, we conducted a comprehensive literature review on each treatment type for cHCC-CCA to capture all relevant studies reporting on treatment efficacy and post-treatment outcomes. Outcomes of interest were response rate and overall survival (OS), disease-free survival (DFS), and progression-free survival (PFS). Reviews on cHCC-CCA and cHCC-CCA treatment were screened for additional studies. Reviewed treatments included liver resection (LR), liver transplantation (LT), ablation (radiofrequency [RFA] and microwave [MWA]), transarterial chemoembolisation (TACE), internal radiotherapy (In-RT), external radiotherapy (Ex-RT), hepatic arterial infusion chemotherapy/pump (HAIP), systemic chemotherapy (CTx), and immunotherapy. The contextualisation, including the literature-reported outcomes per treatment type, has been covered in the discussion section.

### Statistical analysis

Descriptive statistics were used to summarise the results. Variables were expressed as numbers and percentages (%) or as medians and interquartile ranges (IQRs). All statistical analyses and data visualisations were performed using R Core Team (2013) (R Foundation for Statistical Computing v4.1.1, 2021-08-10, Vienna, Austria).

## Results

The survey was distributed to 537 recipients. Of these, 195 recipients were non-patient-treating clinicians or full-time researchers, who were not part of the target population of this study. The remaining 342 recipients were treating clinicians and included 92 surgeons, 57 hepatologists/gastroenterologists, and 148 oncologists. Eventually, a total of 155 surveys were recorded between 23 June and 11 November 2021. Fifty-eight surveys (37%) were partially completed and were therefore excluded. Of the remaining 97 fully completed surveys, one was completed by a non-patient-treating clinician and one by a full-time researcher, and were therefore excluded from the final study cohort. In addition, eight duplicate records were removed. The final number of surveys analysed was 87, representing 56% of the total number of surveys recorded and 25% of the total number of surveys distributed to treating clinicians.

### Respondent demographics and centre specifics

**Fig. 1:**
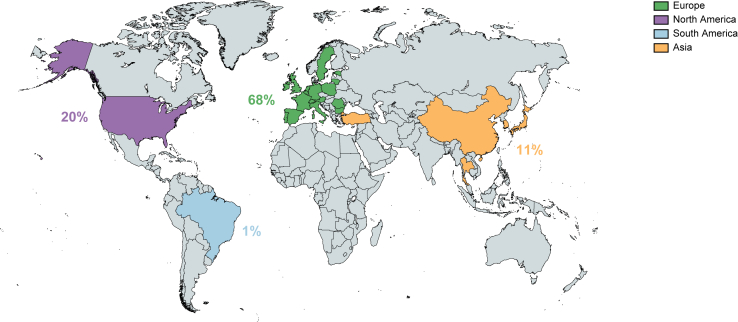
Demographics of respondents.

The respondents covered four continents: Europe (68%), North America (20%), Asia (11%), and South America (1%) (Fig. 1). The number of respondents per country, from highest to lowest, were: USA (17, from 13 different states), Italy (13), Spain (11), Germany (7), France (4), UK (4), Austria (3), South Korea (3), The Netherlands (3), Romania (3), Japan (2), Poland (2), Switzerland (2), China (2), Belgium (1), Brazil (1), Bulgaria (1), Estonia (1), Ireland (1), Lithuania (1), Portugal (1), Singapore (1), Sweden (1), Thailand (1), and Turkey (1).

Participant roles included surgeon (46%, n = 40), oncologist (29%, n = 25), and hepatologist/gastroenterologist (25%, n = 22). The medical specialties they represented were most experienced with treating HCC, although 62% (n = 54) indicated that any type of primary liver cancer is treated by their department (Table S1). Over the past 5 years, the majority (53%) of departments saw more than 80 new patients with primary liver cancer annually, whereas 25% saw more than 140 new primary liver cancer patients annually (median 200, IQR 170–235). A wide variety of treatment modalities were available at each respondent’s centre, of which LR, ablation, TACE, and CTx were the most common, whereas LT was available in 70% of the centres (Table S2).

### cHCC-CCA incidence and pre-treatment diagnosis rate

All but one respondent reported that their department had seen at least one new patient with cHCC-CCA in the past 5 years, where two-thirds had seen at least six to ten new patients with cHCC-CCA (Table 1). The reported proportion of patients with cHCC-CCA diagnosed as such before treatment was a median of 30% (IQR 5–50%). However, this varied by specialty: surgery 10% (IQR 4–29%), hepatology/gastroenterology 30% (IQR 10–60%), oncology 50% (IQR 30–83%).

**Table 1:** **Total number of new patients with cHCC-CCA seen at the respondents’ department in the past 5years**.

Number of new patients with cHCC-CCA seen	Total (N = 87)	Hepatologist/gastroenterologist (n = 22)	Oncologist (n = 25)	Surgeon (n = 40)
0	1 (1%)	0 (0%)	0 (0%)	1 (2%)
1–5	29 (33%)	11 (50%)	7 (28%)	11 (28%)
6–10	25 (29%)	6 (27%)	6 (24%)	13 (32%)
11–15	12 (14%)	3 (14%)	4 (16%)	5 (12%)
16–20	6 (7%)	1 (5%)	2 (8%)	3 (8%)
21–25	5 (6%)	0 (0%)	3 (12%)	2 (5%)
26–30	2 (2%)	0 (0%)	0 (0%)	2 (5%)
31–35	3 (3%)	0 (0%)	2 (8%)	1 (2%)
>35	4 (5%)	1 (5%)	1 (4%)	2 (5%)

cHCC-CCA, combined hepatocellular-cholangiocarcinoma.

### Long-term outcomes of patients with cHCC-CCA

The general perception of respondents was that patients with cHCC-CCA have worse survival (76%, n = 65) and higher recurrence rates (59%, n = 51) than patients with pure HCC, but similar survival (56%, n = 48) and recurrence (51%, n = 44) as patients with CCA (Table S3).

### Patient-specific treatment recommendations for cHCC-CCA

#### Scenario 1 – singular: a segment 3 lesion of 2.0, 3.2, 4.8, or 6.0 cm

**Fig. 2:**
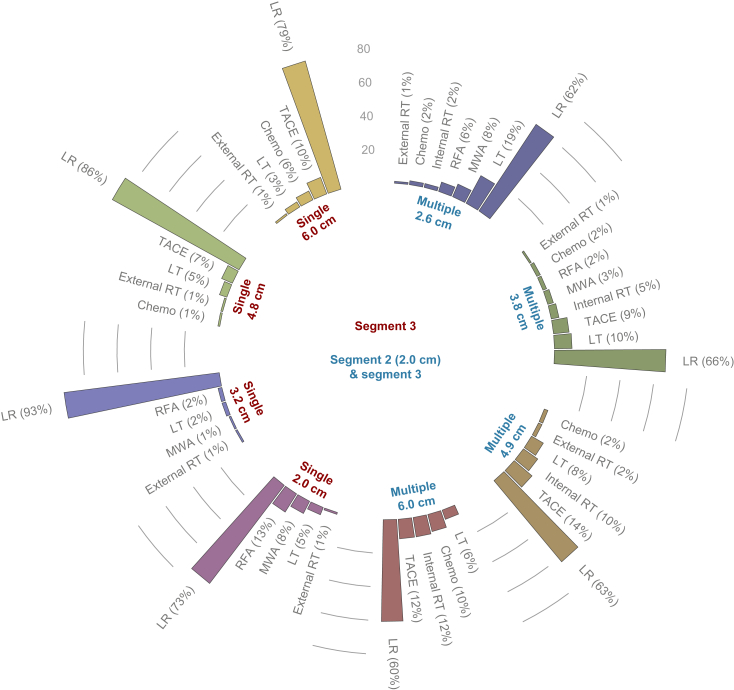
Treatment proposals of the total cohort of respondents. LR, liver resection; LT, liver transplantation; MWA, microwave ablation; RFA, radiofrequency ablation; RT, radiotherapy; TACE, transarterial chemoembolisation.

For each of the single lesion sizes, the majority recommended LR as a first treatment approach (73–93%), followed by ablation for the sizes 2.0 cm and 3.2 cm, and TACE for lesions sized 4.8 cm and 6.0 cm (Fig. 2). LT was proposed by only 2–5% of all clinicians (Fig. 2).

**Fig. 3:**
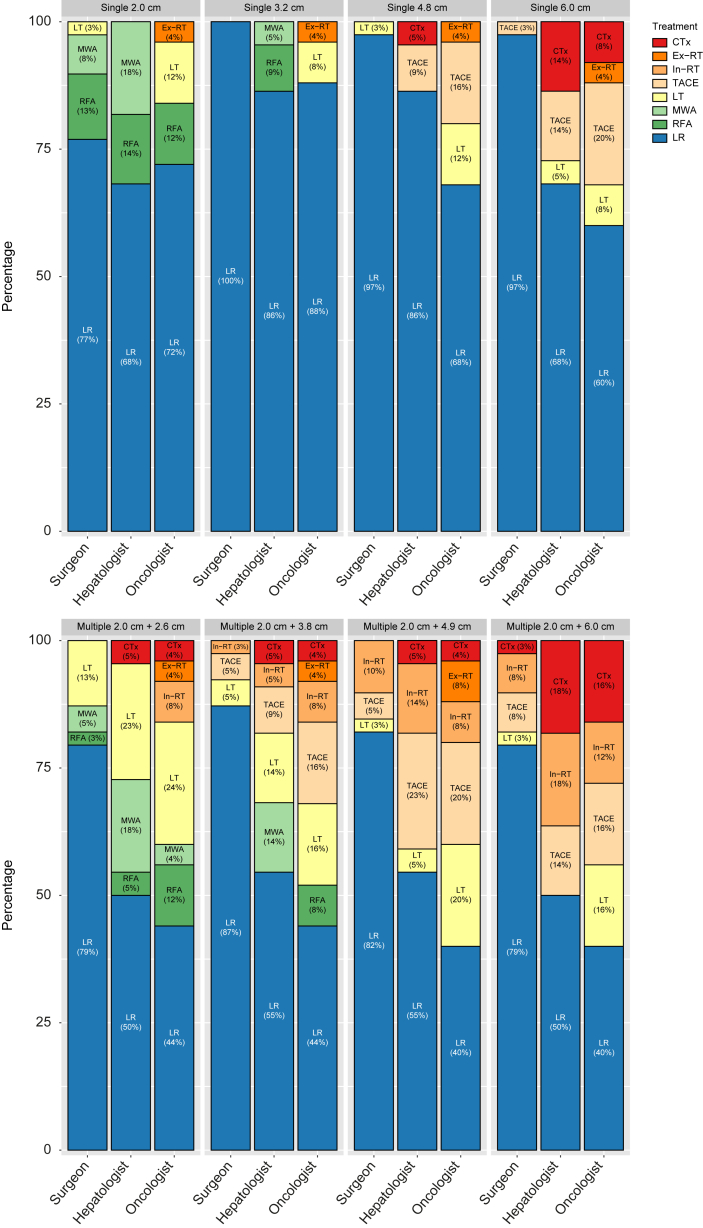
Treatment proposals per specialty of respondents. CTx, systemic chemotherapy; Ex-RT, external radiotherapy; In-RT, internal radiotherapy; LR, liver resection; LT, liver transplantation; MWA, microwave ablation; RFA, radiofrequency ablation; TACE, transarterial chemoembolisation.

Treatment proposals across medical specialties differed. Surgeons consistently recommended LR more often (77–100%) than hepatologists/gastroenterologists (68–86%) and oncologists (60–88%). On the contrary, oncologists more often proposed LT (8–12%) than surgeons (0–3%) and hepatologists/gastroenterologists (0–5%) (Fig. 3). Whether LT was available as treatment at the respondent’s centre did not change these outcomes. For 4.8 cm and 6.0 cm lesions, surgeons still mainly opted for surgical interventions (97–100%), whereas hepatologists/gastroenterologists more often switched to TACE (9–14%) and chemotherapy (5–14%), and oncologists to TACE (16–20%), Ex-RT (4%), and chemotherapy (0–8%) (Fig. 3).

#### Scenario 2 – multifocal: a segment 2 lesion of 2.0 cm and a segment 3 slesion of 2.6, 3.8, 4.9, or 6.0 cm

When two lesions were present, one of 2.0 cm and the other ranging between 2.6 and 6.0 cm, LR was the treatment of choice for most clinicians (60–66%) (Fig. 2). With a second lesion size of 2.6 cm, LR was the preferred treatment was followed by LT (19%) and ablation (14%) (Fig. 2). For a 3.8 cm size, LT (10%) and TACE (9%) were the second and third most proposed treatment strategies (Fig. 2). With a larger second lesion size of 4.9 cm and 6.0 cm, besides TACE (12–14%), In-RT became more pronounced as the proposed alternative treatment option (10–12%), with LT still being proposed by 6–8% of the respondents (Fig. 2).

Also, in this scenario of two lesions (one static of 2.0 cm and one varying between 2.6 and 6.0 cm), surgeons consistently proposed LR in higher rates (79–87%) than hepatologists/gastroenterologists (50–55%) and oncologists (40–44%) (Fig. 3). Moreover, LT was still most often proposed by oncologists (16–24%), followed by hepatologists/gastroenterologists (0–23%), and least often by surgeons (3–13%) (Fig. 3). LT treatment availability in the respondent’s centre did not cause any major changes in how these outcomes related to one another. Overall, more than 80% of the surgeons adhered to surgical intervention as the recommended treatment, regardless of the size of the second lesion. Hepatologists/gastroenterologists and oncologists, however, were progressively less likely to propose surgery as an intervention as the second lesion increased in size, with only 50% of the hepatologists/gastroenterologists and 56% of the oncologists when the second lesion measured 6.0 cm (Fig. 3). For such a patient with a 2.0 and 6.0 cm lesion, hepatologists/gastroenterologists would instead more often recommend chemotherapy (18%), In-RT (18%), and TACE (14%). Similarly, oncologists would more often propose chemotherapy (16%), TACE (16%), and In-RT (12%) (Fig. 3).

If the grade of tumour differentiation in the hypothetical case would have been moderately differentiated instead of well-differentiated, 85% of the clinicians would maintain their initially proposed treatment strategies in all scenarios (surgeons 87%, hepatologists/gastroenterologists 92%, oncologists 73%). However, if the tumour was poorly differentiated, 45% would have made different treatment propositions (surgeons 49%, hepatologists/gastroenterologists 36%, oncologists 50%).

### LT as treatment option for cHCC-CCA

Fifty-one of the 86 clinicians (59%) would consider LT as a treatment option for a biopsy-proven patient with cHCC-CCA (surgeons [64%], hepatologists/gastroenterologists [55%], oncologists [56%]). In this consideration of LT, most clinicians accepted a single lesion size limit of 2 cm (14%), 3 cm (26%), or 5 cm (49%), although three respondents also mentioned an accepted limit of 10 cm (6%). Of the LT-considering respondents, 33 (65%) would also consider LT as a treatment option for patients with multiple cHCC-CCA lesions. A combination of three lesions with a maximum largest lesion size of 3 cm was the accepted limit for most of these clinicians (21 [64%]). Other limit combinations mentioned were three lesions with a maximum largest lesion size of 2 cm (6%) or 5 cm (12%), and five lesions with a maximum size of 3 cm (6%) or 5 cm (6%). Twenty-eight of the clinicians considering LT a viable treatment option (55%) would only consider it if the tumour was well-differentiated, whereas 14 (27%) would consider it even if the tumour was moderately differentiated. Only nine clinicians (18%) would still consider LT a feasible treatment option if the tumour was shown to be poorly differentiated.

### cHCC-CCA treatment policy

Only 11 of the 86 responding clinicians (13%) indicated that a treatment policy for cHCC-CCA exists in their centre. However, most of these policies were non-specific and focused not so much on the exact treatment approach but more on how to interpret the behaviour of this tumour. Reported policies typically comprised multidisciplinary team discussions (three [27%]) and a treatment approach as per HCC or iCCA guidelines based on clinicopathological features and behaviour (four [36%]), where some explicitly report LT should (two [18%]) or should not (two [18%]) be considered. Two of the 11 policies (18%) described a treatment strategy where LR is preferred, whereas one policy also states that, if feasible, downstaging to LR or LT should be considered.

## Discussion

In this study, we evaluated the management of cHCC-CCA from an international, multidisciplinary cohort of clinicians working in large primary liver cancer-treating centres. Except for one, all departments of the 87 unique respondents, covering four continents, had seen at least one to five new patients with cHCC-CCA at their centre in the past 5 years. Overall, most respondents preferred liver resection as the first treatment approach, although marked interdisciplinary differences within each scenario existed. Surgeons mainly adhered to a surgical approach, whereas up to half of the hepatologists/gastroenterologists and oncologists switched to alternative treatment options as tumour burden increased. Interestingly, liver transplantation as a treatment option was most often suggested by oncologists and least by surgeons. Nonetheless, a similarly high proportion of clinicians across medical specialties would consider liver transplantation as a viable treatment option for their patients with cHCC-CCA, with the Milan criteria (single lesion ≤5 cm, or two to three lesions ≤3 cm) reported as the most acceptable limit. The availability of cHCC-CCA treatment policies was limited and policies were often non-specific.

Diagnosing cHCC-CCA before treatment initiation has proved to be difficult as diagnostic imaging by computed tomography (CT) or magnetic resonance imaging (MRI) has a sensitivity of only 5.7–7.1% because of the high degree of tumour heterogeneity.^11^^,^^12^ In theory, biopsies can significantly increase the number of pretreatment diagnoses, with a reported sensitivity of 33% without and 48% with additional immunostaining, were it not for the fact that this procedure is relatively rarely performed.^13^ The main reason for this is that most patients are initially misdiagnosed as having HCC, which typically does not require additional diagnostics beyond confirmation via CT or MRI.^11^^,^^14^^,^^15^ One of the few exceptions to this is the start of systemic treatment, for which additional biopsy is usually performed to confirm the type of malignancy. This was also reflected in our study, where the experienced pre-treatment cHCC-CCA diagnosis rate was generally low (median 30%), although highest in the group of oncologists (up to 100% in some centres). The great potential of biopsy in cHCC-CCA diagnostication has also been highlighted in a recent study by Calderaro *et al.* which demonstrated that nestin, a progenitor cell marker, has strong diagnostic discriminatory power to distinguish cHCC-CCA from HCC (AUC 0.85, sensitivity 0.75, specificity 0.93). Moreover, the authors in this study showed that high nestin levels (>30% neoplastic cells with positive staining) were associated with worse OS and DFS.^16^ Another promising tool that may contribute to improved future identification of cHCC-CCA tumours, especially in the determination of extrahepatic spread, is ^18^F-fluorodeoxyglucose positron emission tomography/computed tomography (^18^F-FDG PET/CT). Previous studies have shown cHCC-CCA tumours to have high FDG-avidity (≥80%), making this imaging modality an interesting complement to the standard staging CT.[17], [18], [19], [20]

Given the low sensitivity of diagnosing cHCC-CCA on imaging and the absence of a standard biopsy, the diagnosis of cHCC-CCA is made primarily by histopathological assessment of surgically resected tissue.^11^^,^^12^^,^^14^^,^^15^ Consequently, most experience with cHCC-CCA has been derived from patients treated with hepatic resection. OS outcomes post-resection range widely but have been reported up to a median of 60 months, 77.5% at 3 year, and 64.4% at 5 year, whereas reported DFS rates do not exceed 38.7% at 5 year.^14^^,^[21], [22], [23], [24] As these are acceptable outcomes, experience with other treatments is limited, and resection has curative potential, LR is usually considered the first-line treatment in patients with resectable cHCC-CCA. Our respondents corroborated this approach, as in each of the different scenarios, the majority opted for LR as the initial treatment. However, when looking at each specialty separately, it was seen that with increasing tumour burden, hepatologists/gastroenterologists and oncologists tended more toward non-surgical treatments such as TACE, radiotherapy, and chemotherapy, whereas most surgeons adhered to LR as the preferred treatment regardless of tumour number and size.

Interestingly, compared with surgeons, hepatologists/gastroenterologists and oncologists also considered LT more often. Comparing LT with LR for patients with cHCC-CCA, previous studies show no statistically significant difference in long-term survival and disease-free outcomes.^12^^,^^21^^,^[24], [25], [26], [27] Hence, a clear rationale to opt for transplantation over resection in resectable patients is missing. However, a recently published study on cHCC-CCA comparing 99 LT patients with 109 LR patients, all treated between 2009 and 2017, shows that both 5-year OS and DFS rates are significantly higher after LT.^14^ When stratified according to Milan and UCSF criteria, only DFS for patients within Milan criteria remained statistically significantly different at 5-year post-treatment (LT 70.1% *vs.* LR 33.6%).^14^ Notwithstanding this, several studies looking at LT outcomes in patients with cHCC-CCA show promising results, reaching 5-year post-transplant survival rates exceeding 65%.^14^^,^^24^^,^^28^^,^^29^ Most of the patients included in these studies had a tumour burden within Milan criteria, a limit that was also considered the optimal cut-off for contemplating LT by the respondents in our study. Nestin can potentially further aid in the future selection of patients most likely to benefit from LT as Calderaro *et al.* have shown that patients who underwent transplantation and had cHCC-CCA and low levels of nestin (≤30% neoplastic cells with positive staining) can achieve a favourable 5-year OS of ∼70%.^16^

A considerable number of respondents in our study (14-21%) would propose ablation for cHCC-CCA lesions sized less than 3 cm. An interesting observation as there are very few studies reporting on outcomes after ablation for patients with cHCC-CCA. The only study with a sizable number of patients with HCC-CCA who underwent ablation (*i.e.* >10) is based on data from the National Cancer Data Base (NCDB) and contains 77 patients who underwent ablation but whose size and the number of lesions are not explicitly reported.^30^ The median survival in this group is ∼15 months, whereas survival rates at 3 and 5 year are ∼35% and 19%, respectively.^30^ Another study looking at 10 patients with cHCC-CCA with unspecified tumour load (three treatment-naive, seven with recurrence after surgical resection) treated with ablation in a single Chinese centre reports a median post-ablation survival of 10 months in the treatment-naive patient group and 20 months in the recurrence group.^31^ Both groups demonstrate a 3-year OS of ∼35%.^31^ DFS, only reported for the treatment-naive group, was a median of 7.2 months.^31^ Lastly, a national database study from Japan studied the short-term radiologic treatment effect of RFA in six patients with either one or two cHCC-CCA lesions sized ≤3 cm.^32^ The technical success rate post-RFA was 100%. However, after 3 months, two of the six patients already showed disease progression.^32^ What can be inferred from these results is that, based on the literature, outcomes after ablation appear less promising than those after surgery, giving cause for caution when considering the use of ablation in patients with resectable cHCC-CCA tumours.

In the setting of more advanced tumour burden, TACE was increasingly suggested as the preferred treatment by the respondents of our study. Varying from 7% to 14% for patients with lesions of 3.8–6.0 cm. In the few studies reporting on survival outcomes in patients with cHCC-CCA treated with TACE, median survival ranges mainly between 6.0 and 12.3 months, and 1- and 3-year survival between 46–52% and 10–16%, respectively.^31^^,^^33^^,^^34^ The study with the largest sample size has been conducted in South Korea and analysed a heterogeneous group of 50 histopathologically confirmed patients with cHCC-CCA.^34^ This single centre shows a post-TACE median survival of 12.3 months and a 1-, 2-, 3-, 4-, 5-year OS of 52%, 38%, 16%, 12%, and 0%, respectively.^34^ After stratification into subgroups, they demonstrate favourable median survival rates in patients with tumours sized ≤9 cm compared with >9 cm (21.2 *vs.* 5 months), patients with Child–Pugh A compared with B (21.2 *vs.* 6.2 months), patients without portal vein invasion compared with patients with portal vein invasion (21.2 *vs.* 4 months), and patients with hypervascular lesions compared to hypovascular lesions on the arterial phase CT scans (16 *vs.* 4 months).^34^ All were confirmed as being independent risk factors in their multivariable analysis.^34^ In addition, another study in the same centre shows that patients with cHCC-CCA with global arterial enhancement (>50% of the tumour volume) achieve better results than patients with cHCC-CCA with only peripheral/rim enhancement or iso-enhancement (median survival of 52.8 *vs.* 12.4 months, respectively).^35^ When evaluating post-TACE treatment response in patients with cHCC-CCA, only few demonstrate complete response (0–12.5%).[32], [33], [34]^,^^36^ Partial response was seen in 8.3–70%, stable disease in 8–62.5%, and disease progression in 10–62%.[32], [33], [34]^,^^36^ In terms of disease control, defined as a complete or partial response or stable disease, a rate of 38–90%.[32], [33], [34]^,^^36^ Reported median PFS post-TACE, however, does not exceed 2.3 months.^31^^,^^33^ TACE thus appears to provide meaningful survival prolongation in selected patients with cHCC-CCA, but limited potential in effectively stabilising tumour growth.

Whereas evaluation of external radiotherapy in patients with cHCC-CCA is completely lacking, some results are known about the effect of internal radiotherapy. In our study the second most frequent non-surgical treatment recommended for patients with multiple nodules (2–12%). The studies looked at the effect of transarterial radioembolisation (TARE) with yttrium-90 in heterogeneous groups of patients with cHCC-CCA, ranging between 10 and 22 patients, and demonstrated median survival rates that lie between 9.3 and 15.2 months.[37], [38], [39] Although the radiological response rate after TARE varies widely with a complete response of 0–60%, a partial response of 10–60%, stable disease of 0–40%, and disease progression of 0–50%, the disease control rate shows a potential beneficial effect ranging between 50 and 100%.[36], [37], [38], [39] Translated into PFS, lying between 5.2 and 16.6 months on the median.^37^^,^^39^

Hepatic arterial infusion chemotherapy (HAIC) was not suggested by any respondent in our study. A lack of supporting evidence may explain this. The only multi-case study reporting on outcomes after HAIC treatment in patients with cHCC-CCA analysed six patients that received HAIC through a pump (also known as HAIP).^36^ Five of them received floxuridine, one floxuridine plus gemcitabine.^36^ Four out of six patients showed partial response, where two were even downstaged to a state in which they could be resected.^36^ The remaining two patients treated showed stable disease.^36^ Unfortunately, no long-term outcomes were reported for these patients.

Systemic therapy was rarely suggested in our study. Even with tumours of 6.0 cm, the proportion of respondents opting for systemic therapy did not exceed 10%. Systemic therapy in patients with cHCC-CCA has primarily been evaluated in heterogeneous groups that received either sorafenib or a combination of gemcitabine, fluorouracil, and platinum drugs (cisplatin or oxaliplatin).^31^^,^^36^^,^[40], [41], [42], [43] Most results are known for the combination of gemcitabine and platinum drugs and show a median survival of 10.2–16.2 months with 1- and 2-year OS rates of 66% and 26.1%.[40], [41], [42] Median PFS ranged between 3.0 and 9.0 months with 1- and 2-year PFS rates of 24.2% and 9.7%.[40], [41], [42] Disease control rates reported in these populations ranged between 78.4 and 78.6%.^40^^,^^41^ Evaluation of gemcitabine ± fluorouracil has shown similar survival outcomes (median OS 11.7 months, median PFS 6.6 months), although its disease control rate was less (38.5%) than seen after gemcitabine/platinum.^40^ Another combination therapy with comparable results is that of fluorouracil with cisplatin, showing a median survival of 11.9 months and a median PFS of 3.8 months.^42^ A significantly different result is seen after treatment with sorafenib, showing a median survival of only 3.5–9.6 months, a median PFS of 1.6–4.8 months, and a disease control rate of no more than 20%.^40^^,^^42^ As such, the effects of systemic therapy in patients with cHCC-CCA seems to be more consistent with that of patients with CCA rather than patients with HCC.

Although the role of immunotherapy for cHCC-CCA is limited to two case reports, both show encouraging results.^44^^,^^45^ The first case showed a complete radiological remission of six pulmonary metastasis 9 months after starting pembrolizumab with the patient still being recurrence-free 33 months after treatment-start.^44^ The second case was treated with ipilimumab and nivolumab, followed by nivolumab, and showed a radiological near-complete response of multiple intrahepatic lesions 11 months after treatment initiation.^45^ Results that argue for including patients with advanced cHCC-CCA in future immunotherapy studies.

At the moment of writing, only two working groups have published recommendations for treating patients with cHCC-CCA.^46^^,^^47^ The rare liver tumours working group of the European Reference Network on Hepatological Diseases (ERN RARE-LIVER) concluded that an evidenced-based management approach for the systemic treatment of cHCC-CCA is currently still not available.^46^ A working group report from the ILTS Transplant Oncology Consensus Conference, mainly focused on LT as a treatment for cHCC-CCA, concluded that there is still no consensus about the actual role of LT in the therapeutic algorithm of cHCC-CCA tumours.^47^ The lack of policies in our respondents’ centres is therefore not surprising as the literature does not corroborate the opportunity to derive a clear treatment policy for cHCC-CCA. Given the marked interdisciplinary difference in treatment propositions observed in our study, where the received treatment is highly dependent on the type of specialist treating the patient instead of on the explicit tumour load of a patient, there is a strong need for an unambiguous treatment policy for patients with cHCC-CCA.

In brief, the literature suggests that surgical intervention provides the most encouraging long-term results, whereas ablation seems a suitable alternative in case of irresectability. When these treatments are not an option, TACE, internal radiotherapy (TARE), and systemic therapy seem to be equally effective. However, in the latter it seems prudent to consider sorafenib as a last resort as it seems to have an inferior effect compared with the rest. Finally, not much can be concluded on the use of HAIC/HAIP and immunotherapy, although the few studies available demonstrate a potentially promising effect. In contrast, no statements can be made about external radiotherapy owing to a complete absence of literature about it for patients with cHCC-CCA. Considering this and acknowledging our study results, it seems that some hepatologists/gastroenterologists and oncologists might be practicing a too conservative treatment strategy as the literature suggests that a surgical intervention should always be considered as a first approach in patients deemed resectable.

### Limitations

Inextricably related to a survey-based design, there might be the potential for non-response bias. Although our respondents might not represent the experience and policy of every centre and country worldwide, our results nonetheless reflect a demographically highly diverse response from four different continents, 25 different countries, and four different specialties working in medium to large primary liver-cancer treatment centres. As surgeons were relatively overrepresented compared with hepatologists/gastroenterologists and oncologists, the aggregate results may be skewed. To account for this, outcomes were also stratified by specialty. As with each survey study, it is subject to recall bias, and there is a potential for discrepancy between reported experiences and actual decisions made. This was reduced by limiting the number of questions about past experiences and focusing on the respondent’s contemporary (*i.e.* prospective) decision-making process, reflecting the unbiased translation of professional knowledge into future decision-making. To capture this, we presented our respondents with only eight different scenarios, whereas clinically, many more can occur, limiting the clinical generalisability of our results. Nonetheless, this is the first survey mapping the current hospital-wide approach to cHCC-CCA, providing insights that may spur the development of therapeutic guidelines for cHCC-CCA.

### Conclusions

The international community, represented by surgeons (n = 40), hepatologists/gastroenterologists (n = 25), and oncologists (n = 22), favoured LR as the first treatment approach to cHCC-CCA, with most clinicians also agreeing that LT should be considered. Notwithstanding this, marked interdisciplinary differences in treatment strategies existed where the received treatment is highly dependent on the type of specialist treating the patient instead of on the explicit tumour load of a patient. The lack of institutional cHCC-CCA treatment protocols undoubtedly contributed to this discordance, expressing the need for an unambiguous treatment policy for patients with cHCC-CCA.

## Financial support

This study received no financial support.

## Authors’ contributions

Conception of project: MC, TI, BB, RdW, WP, GS, JIJ. Literature review: MC, TI, WP, GS, JIJ.

Data analysis: MC. Interpretation results: MC, TI, BB, RdW, WP, GS, JIJ. Write up of the manuscript: MC, TI, BB, RdW, WP, GS, JIJ. All authors have given final approval for this manuscript to be submitted to *JHEP Reports*.

## Data availability statement

All data generated or analysed during this study are included in this article and its supplementary material files. Individual participant data are available upon reasonable request to the corresponding author.

## Conflicts of interest

GS discloses consultancy for AstraZeneca, Roche, Novartis, Evidera and Integra. He has also received financial compensation for talks for Roche, AstraZeneca, Chiesi, and Integra, and has received a grant from Roche. None of the other authors have any conflicts of interest to declare.

Please refer to the accompanying ICMJE disclosure forms for further details.
