# Supplementary material for: An international multicentre evaluation of treatment strategies for combined hepatocellular-cholangiocarcinoma✰
Source: JHEP Rep. 2023 Mar 28;5(6):100745. doi: 10.1016/j.jhepr.2023.100745 (PMC10206495; doi:10.1016/j.jhepr.2023.100745)
Supplement: Multimedia component 1 [file mmc1.pdf]

An international multicenter evaluation of treatment strategies for combined hepatocellular-cholangiocarcinoma

Marco P.A.W. Claasen, Tommy Ivanics, Berend R. Beumer, Roeland F. de Wilde, Wojciech G. Polak, Gonzalo Sapisochin, Jan N.M. IJzermans

Table of contents

Table S1 2

Table S2 2

Table S3 3

Full survey 4

Table S1. Primary liver cancer tumor types treated by the respondents' specialty

Primary liver cancer tumor type	Total (N=87)	Hepatologist/ gastroenterologist (N=22)	Oncologist (N=25)	Surgeon (N=40)
Hepatocellular carcinoma (HCC)	84 (97%)	20 (91%)	24 (96%)	40 (100%)
Intrahepatic cholangiocarcinoma (iCCA)	81 (93%)	19 (86%)	24 (96%)	38 (95%)
Perihilar cholangiocarcinoma (pCCA)	78 (90%)	17 (77%)	24 (96%)	37 (92%)
Fibrolamellar hepatocellular carcinoma (FL-HCC)	65 (75%)	10 (45%)	20 (80%)	35 (88%)
Combined hepatocellular-cholangiocarcinoma (cHCC-CCA)	77 (89%)	17 (77%)	23 (92%)	37 (92%)
Hepatic epithelioid hemangioendothelioma (HEHE)	63 (72%)	12 (55%)	16 (64%)	35 (88%)
Liver neuroendocrine tumours (Liver NET)	70 (80%)	14 (64%)	19 (76%)	37 (92%)

Table S2. Treatment modalities for primary liver cancer available at the respondents' center

Treatment modality	Total (N=87)	Hepatologist/ gastroenterologist (N=22)	Oncologist (N=25)	Surgeon (N=40)
Liver resection (LR)	83 (95%)	20 (91%)	23 (92%)	40 (100%)
Liver transplantation (LT)	61 (70%)	12 (55%)	15 (60%)	34 (85%)
Radiofrequency ablation (RFA)	79 (91%)	18 (82%)	23 (92%)	38 (95%)
Microwave ablation (MWA)	74 (85%)	18 (82%)	21 (84%)	35 (88%)
Transarterial chemoembolization (TACE)	80 (92%)	20 (91%)	21 (84%)	39 (98%)
Internal radiotherapy (In-RT)	57 (66%)	11 (50%)	18 (72%)	28 (70%)
External radiotherapy (Ex-RT)	66 (76%)	12 (55%)	22 (88%)	32 (80%)
Hepatic artery infusion pump (HAIP)	26 (30%)	2 (9%)	13 (52%)	11 (28%)
Systemic chemotherapy (CTx)	84 (97%)	20 (91%)	25 (100%)	39 (98%)

Table S3. Outcomes cHCC-CCA patients compared to patients with either HCC or CCA

	Total (N=86)	Hepatologist/ gastroenterologist (N=22)	Oncologist (N=25)	Surgeon (N=39)
**Survival cHCC-CCA compared to HCC**				
Better survival	5 (6%)	0 (0%)	4 (16%)	1 (3%)
Similar survival	10 (12%)	1 (5%)	3 (12%)	6 (15%)
Worse survival	65 (76%)	18 (82%)	16 (64%)	31 (79%)
Do not know	6 (7%)	3 (14%)	2 (8%)	1 (3%)
**Survival cHCC-CCA compared to CCA**				
Better survival	17 (20%)	4 (18%)	3 (12%)	10 (26%)
Similar survival	48 (56%)	13 (59%)	13 (52%)	22 (56%)
Worse survival	13 (15%)	2 (9%)	7 (28%)	4 (10%)
Do not know	8 (9%)	3 (14%)	2 (8%)	3 (8%)
**Recurrence cHCC-CCA compared to HCC**				
Higher recurrence	51 (59%)	14 (64%)	12 (48%)	25 (64%)
Similar recurrence	18 (21%)	3 (14%)	4 (16%)	11 (28%)
Lower recurrence	5 (6%)	1 (5%)	3 (12%)	1 (3%)
Do not know	12 (14%)	4 (18%)	6 (24%)	2 (5%)
**Recurrence cHCC-CCA compared to CCA**				
Higher recurrence	9 (10%)	2 (9%)	3 (12%)	4 (10%)
Similar recurrence	44 (51%)	13 (59%)	14 (56%)	17 (44%)
Lower recurrence	16 (19%)	2 (9%)	2 (8%)	12 (31%)
Do not know	17 (20%)	5 (23%)	6 (24%)	6 (15%)

**CCA: Cholangiocarcinoma, cHCC-CCA: Combined hepatocellular-cholangiocarcinoma, HCC: Hepatocellular carcinoma*

Section A: Consent

A1. Do you give consent to participate in this survey?

The aim of this survey is to map the current hospital-wide approach to combined hepatocellular-cholangiocarcinoma (cHCC-CCA). Therefore, all data obtained as part of this survey will be processed accordingly to make them available for publication in a medical journal.

Personal data will be handled according to the General Data Protection Regulation (GDPR) and the Erasmus Medical Center privacy guidelines. If you have any questions about the processing of personal data or if you would like it to be deleted, please contact m.claasen@erasmusmc.nl.

- Yes ☐
- No ☐

Section B: General questions

B1. In which country do you practice?

- Argentina ☐
- Australia ☐
- Austria ☐
- Belgium ☐
- Brazil ☐
- Canada ☐
- China ☐
- Czech Republic ☐
- Denmark ☐
- Finland ☐
- France ☐
- Germany ☐
- Greece ☐
- Hong Kong ☐
- India ☐
- Ireland ☐
- Italy ☐
- Japan ☐

Korea, Republic of (South Korea) ☐
Luxembourg ☐
Mexico ☐
Netherlands ☐
New Zealand ☐
Norway ☐
Poland ☐
Portugal ☐
Spain ☐
Sweden ☐
Switzerland ☐
Taiwan (Republic of China) ☐
Turkey ☐
United Kingdom ☐
United States ☐
Other ☐

Other

B2. **In which <u>province/state</u> is your medical center located?**

B3. **What is your <u>medical specialty</u>?**

Surgeon ☐
Oncologist ☐
Hepatologist ☐
Gastroenterologist ☐
Other ☐

Other

B4. **At your center, which <u>primary liver cancer (PLC) tumour types</u> does <u>your medical specialty treat</u>?**

- Hepatocellular carcinoma (HCC) ☐
- Intrahepatic cholangiocarcinoma (iCCA) ☐
- Perihilar cholangiocarcinoma (pCCA) ☐
- Fibrolamellar hepatocellular carcinoma (FL-HCC) ☐
- Combined hepatocellular-cholangiocarcinoma (cHCC-CCA) ☐
- Hepatic epithelioid hemangioendothelioma (HEHE) ☐
- Liver neuroendocrine tumour (Liver NET) ☐
- None of the above ☐

B5. **At your center, which <u>treatment modalities</u> for <u>primary liver cancer (PLC)</u> are available?**

- Liver resection (LR) ☐
- Liver transplantation (LT) ☐
- Radiofrequency ablation (RFA) ☐
- Microwave ablation (MWA) ☐
- Transarterial chemoembolization (TACE) ☐
- Internal radiotherapy (e.g. SIRT, TARE, Y-90, etc.) ☐
- External radiotherapy (e.g. SBRT, EBRT, etc.) ☐
- Hepatic artery infusion pump (HAIP) ☐
- Systemic chemotherapy (e.g. Sorafenib, Gemcitabine/Cisplatin, etc.) ☐
- Other, please specify: ☐

Other, please specify:

____

Section C: <u>Incidence & Diagnosis</u>

C1. **Looking back 5 years, what is the <u>average annual number</u> of <u>new patients</u> with primary liver cancer <u>(PLC)</u> seen in <u>your medical specialty</u>?**

The average number of PLC patients seen anually (regardless of subsequent treatment initiation).

0	1-20	21-40	41-60	61-80	81-100	101-120	121-140	>140
☐	☐	☐	☐	☐	☐	☐	☐	☐

C2. **Based on your previous answer, please specify <u>a more matching average annual number</u> of <u>newly seen PLC</u> patients:**

[ ][ ][ ][ ][ ][ ][ ][ ][ ][ ]

C3. **Looking back 5 years, what is the <u>total number</u> of <u>new patients</u> with combined hepatocellular-cholangiocarcinoma <u>(cHCC-CCA)</u> seen in <u>your medical specialty</u>?**

The total number of cHCC-CCA patients seen in the last 5 years (regardless of subsequent treatment initiation).

0	1-5	6-10	11-15	16-20	21-25	26-30	31-35	>35
☐	☐	☐	☐	☐	☐	☐	☐	☐

C4. **Based on your previous answer, please specify <u>a more matching total number</u> of <u>newly seen cHCC-CCA</u> patients:**

[ ][ ][ ][ ][ ][ ][ ][ ][ ][ ]

C5. **What <u>proportion</u> of patients with combined hepatocellular-cholangiocarcinoma <u>(cHCC-CCA)</u> were <u>diagnosed as such before treatment</u>?**

Please try to indicate the proportion as precise as possible. If you really do not know, you can leave it blank.

[ ][ ][ ]

Section D: Outcomes cHCC-CCA patients

Please answer based on your opinion and experience.

D1. **How does <u>overall survival</u> of <u>cHCC-CCA</u> patients <u>compare to similar staged HCC and CCA</u> patients?**

	Better survival	Similar survival	Worse survival	Do not know
Compared to HCC patients	☐	☐	☐	☐
Compared to CCA patients	☐	☐	☐	☐

D2. **How does the <u>recurrence rate</u> of <u>cHCC-CCA</u> patients <u>compare to similar staged HCC and CCA</u> patients?**

HCC Hepatocellular carcinoma CCA Cholangiocarcinoma cHCC-CCA Combined hepatocellular-cholangiocarcinoma

	Lower recurrence	Similar recurrence	Higher recurrence	Do not know
Compared to HCC patients	☐	☐	☐	☐
Compared to CCA patients	☐	☐	☐	☐

Section E: Liver transplantation (LT) for cHCC-CCA

E1. **Would you ever consider LT as a treatment option for a patient with a biopsy proven cHCC-CCA?**

Yes ☐

No ☐

E2. **Which limits would you consider for LT as a treatment option for a patient diagnosed with at least 1 cHCC-CCA lesion?**

	1	2	3	4	5	6	7	8	9	10
Single lesion size limit (cm)	☐	☐	☐	☐	☐	☐	☐	☐	☐	☐
Total number of lesions limit	☐	☐	☐	☐	☐	☐	☐	☐	☐	☐
Multiple lesions size limit of the largest lesion (cm)	☐	☐	☐	☐	☐	☐	☐	☐	☐	☐

E3.

	Well differentiated	Moderately differentiated	Poorly differentiated
Worst tumour differentiation grade	☐	☐	☐

Section F: Treatment decisions cHCC-CCA patients

A 53-year-old female patient with a clean medical history presents itself at your clinic with a biopsy proven well differentiated cHCC-CCA (based on biopsy of the largest lesion). There are no signs of extrahepatic disease or vascular invasion. She has Child-Pugh score A6 and all detected lesions are deemed resectable.

Based on this case, which treatment would you propose for each number/size combination mentioned below?

F1. **A single lesion in segment 3 with a measured size of:**

__LR__ Liver resection Internal RT Internal radiotherapy (e.g. SIRT, TARE, Y-90, etc.) LT Liver transplantation TACE Transarterial chemoembolization MWA Microwave ablation External RT External radiotherapy (e.g. SBRT, EBRT, etc.) RFA Radiofrequency ablation HAIP Hepatic artery infusion pump Chemo Chemotherapy (e.g. Sorafenib, Gemcitabine/Cisplatin, etc.)

	LR	LT	MWA	RFA	Internal RT	TACE	External RT	HAIP	Chemo
2.0 cm	☐	☐	☐	☐	☐	☐	☐	☐	☐
3.2 cm	☐	☐	☐	☐	☐	☐	☐	☐	☐
4.8 cm	☐	☐	☐	☐	☐	☐	☐	☐	☐
6.0 cm	☐	☐	☐	☐	☐	☐	☐	☐	☐

F2. **Multiple lesions: one in segment 2 of 2.0 cm and one in segment 3 with a measured size of:**

	LR	LT	MWA	RFA	Internal RT	TACE	External RT	HAIP	Chemo
2.6 cm	☐	☐	☐	☐	☐	☐	☐	☐	☐
3.8 cm	☐	☐	☐	☐	☐	☐	☐	☐	☐
4.9 cm	☐	☐	☐	☐	☐	☐	☐	☐	☐
6.0 cm	☐	☐	☐	☐	☐	☐	☐	☐	☐

F3. **If the biopsy had shown a moderately differentiated cHCC-CCA lesion instead of a well-differentiated lesion, would this have affected your treatment recommendations?**

Yes ☐

No ☐

F4. **If the biopsy had shown a poorly differentiated cHCC-CCA lesion instead of a well-differentiated lesion, would this have affected your treatment recommendations?**

Yes ☐

No ☐

Section G: Policy cHCC-CCA tumours

G1. **Is there any policy in place in your center regarding the clinical management of combined hepatocellular-cholangiocarcinoma (cHCC-CCA)?**

Yes ☐

No ☐

G2. **Please indicate the decision-making as precisely as possible.**

(Tip: if you click on the text box, you can just start typing)

Section H: Interest participation multicenter study

H1. **Would you be interested in participating in a multicenter retrospective study looking at the demographics and treatment outcomes of combined hepatocellular-cholangiocarcinoma (cHCC-CCA) patients?**

Yes ☐

No ☐

H2. **Please leave your email address here in order for us to contact you.**

Section I: Closure

You answered all questions, please submit the survey via the submission button below.

We want to thank you for your time.

Thank you very much for your time to fill in this survey. Kind regards, Jan IJzermans, Surgeon, Erasmus Medical Center

Gonzalo Sapisochin, Surgeon, Toronto General Hospital

Wojtek Polak, Surgeon, Erasmus Medical Center

Marco Claasen, Research fellow, Erasmus Medical Center

Tommy Ivanics, Research fellow, Toronto General Hospital

Berend Beumer, Research fellow, Erasmus Medical Center

Personal data will be handled according to the General Data Protection Regulation (GDPR) and the Erasmus Medical Center privacy guidelines. If you have any questions about the processing of personal data or if you would like it to be deleted, please contact m.claasen@erasmusmc.nl.
